# Supplementary material for: The SGLT2 Inhibitor Canagliflozin Prevents Carcinogenesis in a Mouse Model of Diabetes and Non-Alcoholic Steatohepatitis-Related Hepatocarcinogenesis: Association with SGLT2 Expression in Hepatocellular Carcinoma
Source: Int J Mol Sci. 2019 Oct 22;20(20):5237. doi: 10.3390/ijms20205237 (PMC6829338; doi:10.3390/ijms20205237)
Supplement: Supplementary file 1 [file ijms-20-05237-s001.pdf]

Supplementary Figure legends

Supplementary Fig.1

a Examination of H-E stained liver sections revealed fatty degeneration, inflammatory cell infiltration, and hepatocellular ballooning, predominantly around the central veins, in mice from the vehicle group
b The NAS score was significantly lower in the canagliflozin group compared with the vehicle group .
c Gene expression of SOCS (suppressor of cytokine signaling) 3 was decreased in the canagliflozin group.
d Sirius red staining showed no significant difference in the collagen deposition area between the canagliflozin group and the vehicle group.
e Expression of type 3 collagen mRNA was significantly lower in the canagliflozin group than in the vehicle group.

Supplementary Fig.2

Expressions of both SGLT1 and SGL2 in HepG2 cells, THP-1 cells, and human umbilical vein endothelial cells (HUVEC).

Supplementary Fig.3

Gene expression of CCND1 (cyclin D1) between treatment with 10 μM canagliflozin and the control

Supplementary Table 1

Body weight and biochemical parameters in NASH mice under diabetic background in Study 1

Supplementary Fig.1

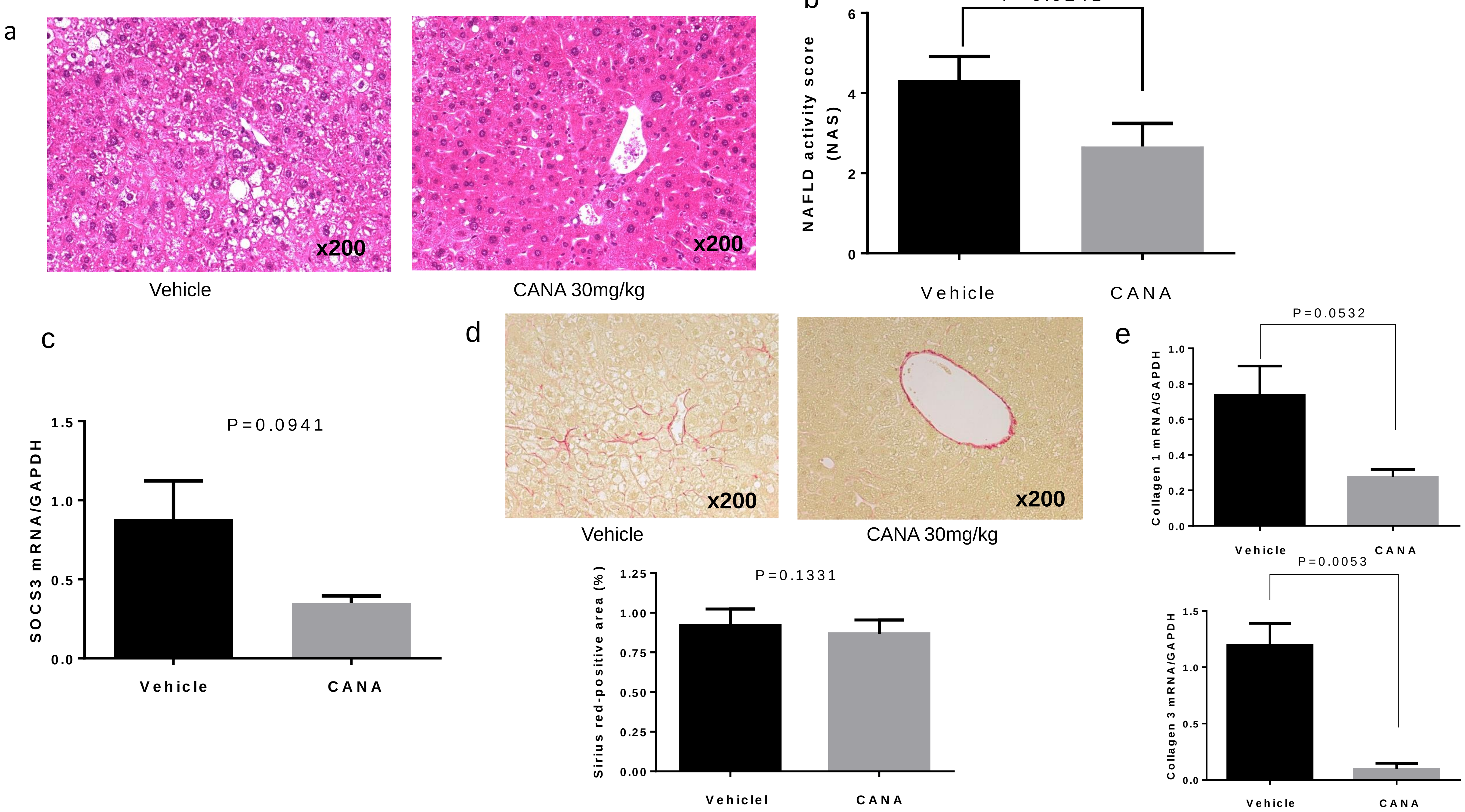

Supplementary Fig.2

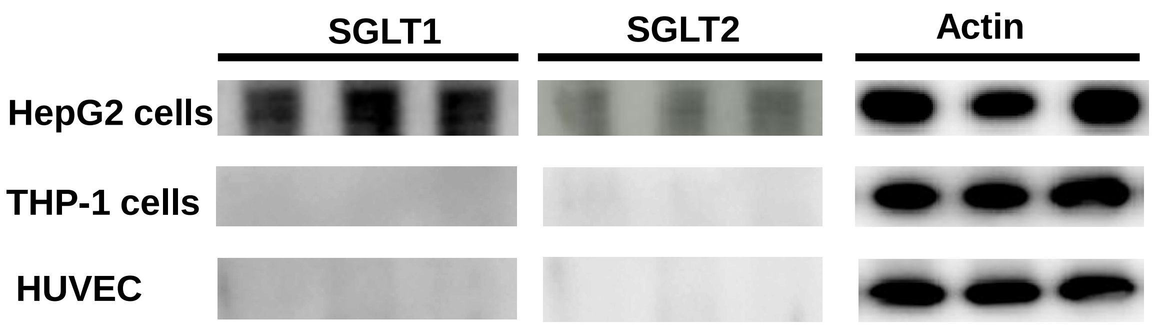

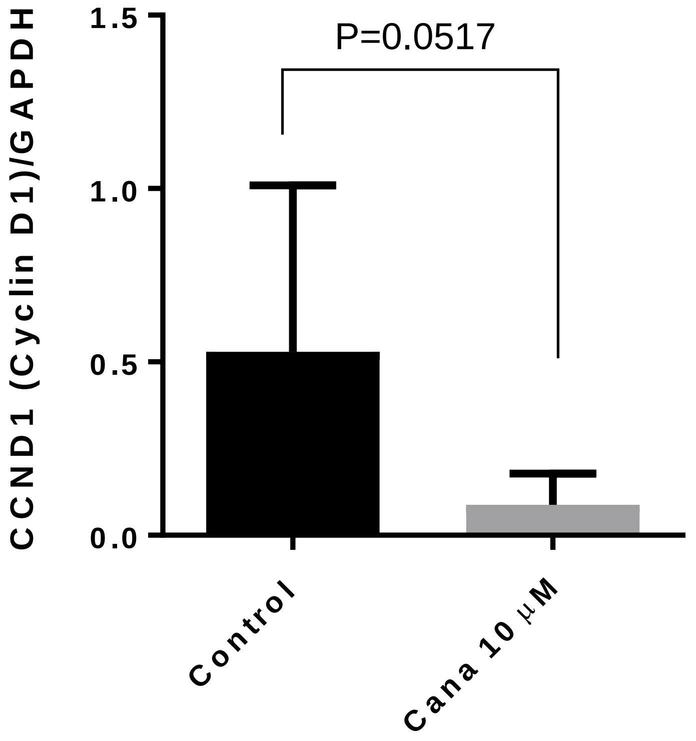

n=6

Supplementary Table 1

Body weight and biochemical parameters in NASH mice under diabetic background

	vehicle (n=3)	Cana 30mg/Kg (n=3)	P-value
Body weight (g)			
Day 0	19.0±1.0	19.0±1.2	0.9726
Day 21	22.7±2.6	21.3±1.3	0.4573
Liver-to-weight ratio (mg/g)	7.0±0.4	5.7±0.3	0.0105
Plasma glucose (mg/dl)	587±43	310±40	0.0012
ALT (U/l)	45.0±4.4	31.7±7.8	0.0605
Triglyceride (mg/dl)	889±193	401±140	0.0240
Serum insulin (pg/ml)	844±556	1018±1372	0.7000

Data are mean±SD. Cana, canagliflozin; ALT, alanine aminotransferase.
